# Austerity, measles and mandatory vaccination: cross-regional analysis of vaccination in Italy 2000–14

**DOI:** 10.1093/eurpub/cky178

**Published:** 2018-09-11

**Authors:** Veronica Toffolutti, Martin McKee, Alessia Melegaro, Walter Ricciardi, David Stuckler

**Affiliations:** 1 “Carlo F. Dondena” Centre for Research on Social Dynamics and Public Policies, Bocconi University, Milan, Italy; 2 Department of Public Health and Policy, London School of Hygiene and Tropical Medicine (LSHTM), London, UK; 3 Department of Social and Political Science, Bocconi University, Milan, Italy; 4 Istituto Superiore di Sanità - National Institute of Health, Rome, Italy

## Abstract

**Background:**

Italy has experienced a resurgence in measles since 2015. Although much emphasis has been placed on the role of individuals opting out of vaccination, here we test the hypothesis that large budget reductions in public health spending were also a contributing factor.

**Methods:**

Multi-variate statistical models were used to assess the relationship between measles, mumps and rubella (MMR) coverage and real public health expenditure per-capita across Italy’s 20 regions covering the period 2000–14.

**Results:**

Between 2010 and 2014 Italy’s public health expenditure fell by over 2%, although varying among regions. Fixed effects models estimate that each 1% reduction in per-capita public health expenditure was associated with a decrease of 0.5 percentage points (95% CI: 0.36–0.65 percentage points) in MMR coverage, after adjusting for time and regional-specific time trends. The consequences can be illustrated by comparing two regions, Lazio, where public health spending fell by 5% and MMR coverage by over 3 percentage points, and Sardinia, a historically deprived region, where public health spending partly rose and MMR rates remained approximately steady.

**Conclusion:**

Adoption of austerity policies in the Italian health system was found to be significantly associated with declining vaccination rates for MMR. However, the recent introduction of mandatory vaccination for Italian children may help counteract this trend.

## Introduction

The adoption of austerity measures in Europe in recent years has, in several cases, impacted adversely on health protection, an area that has often been poorly funded even when economies are performing well.^1^ These measures have been linked to several health damaging effects including suicides,^2^^,^^3^ increase in unmet needs^4^ and disease outbreaks that have disproportionally affected vulnerable groups.^5^ Examples from the most recent global financial crisis include malaria in Greece and HIV in Greece and in Romania,^1^^,^^6^ while, in earlier economic crises, cuts in public health expenditure were linked to outbreaks of diphtheria,^5^ leptospirosis^7^ and tuberculosis.^8^

Several European countries have been experiencing declining vaccination rates and resurgences in measles incidence rates. In 2017 there were over 14 000 incident cases across Europe and 30 fatalities, including 19 in Romania, four in Italy, one in Bulgaria, Germany, Portugal, France and Spain.^9^^,^^10^ Italy has been especially badly affected, with 5004 cases, the second largest number in Europe in 2017.^11–13^

The primary reason for the outbreak is declining uptake of measles vaccination, creating large pockets of measles-susceptible individuals. In Italy, of the incident cases, 88% were never vaccinated and 6% received only one of the two required doses of measles vaccine.^14^ Measles, mumps and rubella (MMR) vaccine coverage is currently below the 95% vaccine coverage target that was set by National Immunization Prevention Plan in 2012 to achieve herd immunity.^15^

There has been widespread debate about how best to respond. Attention has focused on adults who have opted to refuse vaccination for their children (so-called ‘vaccine hesitancy’).^16^ Paolo Gentiloni, the Italian prime minister during the 2017 outbreak, e.g. blamed the return of measles on the ‘spread of anti-scientific theories’. In response, the Italian government implemented a mandatory vaccination policy in 2017. Under the new regime, all children under 16 years are required to have proof of vaccination against 10 common infectious diseases, including measles, prior to enrolment in public schools.

Although, undoubtedly, anti-scientific theories play a crucial role in the potential emergence of new outbreaks, it has been observed that these are more prominent among more affluent households and in the wealthier northern Italian regions.^17^ Yet the increase in measles has been concentrated in more deprived regions and hard to reach populations, suggesting that other factors also came into play.^18–20^ Thus, another view links recent outbreaks to minorities, in particular in Lazio, which experienced one-third of all Italian cases in 2017, where some have drawn connections to the high concentration of Romanian migrants in the region.^21^^,^^22^ This view seemed to be supported by evidence from the outbreaks in 2006 and 2015/16 in the northern regions, the richest part of the country, which were concentrated among Roma and migrants,^19^ although those communities were not especially affected in the 2017 outbreak. In fact, in 2017, 7% of cases occurred in healthcare settings and adults, rather than children, appear to have been most affected.^22^

A further possible factor is the impact of the economic situation on public health capacity. In November 2011, the government reduced the health budget by €7.5 million and introduced co-payments for visits to specialists. The healthcare budget was reduced by €900 million in 2012, a cut of slightly under 1% in a budget that had previously been growing steadily, with a further cut of €1.8 billion in 2013, and then €2 billion more in 2014.^23^ These cuts affected a range of services including prevention programmes, pharmaceuticals, staff and equipment.^24^ Unfortunately it is not possible to isolate specific budget lines in publicly available data; however, official government reports from 2013 to 2014 note that vaccine expenditure dropped by about 10% in this period.^25^

The objective of this study is thus to assess whether austerity in the health sector might have impacted adversely on MMR coverage in Italy.

## Methods

### Data sources

To assess the association between MMR coverage and public health expenditure per-capita, two distinct data sources were linked. The first comprises MMR coverage rates at 24 months of age, so considering the first vaccine dose only, derived from data supplied by the Italian National Institute of Health (Istituto Superiore di Sanità, ISS) for Italy’s 19 regions covering the period 2000–14. The independent province of Trento was considered but Bolzano was excluded from the main analyses because of characteristics that make it an extreme outlier in Italy. This German-speaking province has very low levels of MMR coverage reported (68.8% vs. the Italian average of 85.3%) despite being very wealthy. However, as noted below, it was included subsequently in a series of robustness checks. For the purposes of analysis, we classify the province of Trento as a region for coherence, giving 20 regions in total (Trento and 19 standard regions).

The second dataset provides information on the per-capita annual public health expenditure for the 20 regions, covering the same period, extracted from ‘Health for All’ a database periodically released by the Italian National Institute of Statistics based on a WHO platform.^26^ The public health expenditure was adjusted for inflation using Purchasing Power Parity at 2010 constant prices. Hence we now use the term real public health expenditure. Supplementary Web Appendix table A1 in the Web Appendix 1 provides further details.

### Statistical models

The association between real per-capita public health expenditure and MMR coverage at regional level is analyzed using the following equation:

$${MMR}_{it}=\alpha +{\beta \text{log}(\text{exp}}_{it})+\gamma {year}_{t}+\delta {region}_{i}\cdot {year}_{t}+\varepsilon_{it}$$

where *i* is the region, *t* is the year, *MMR* is the annual coverage level expressed in percentage, *EXP* represents the real public health expenditure per-capita and *ε* represents a random error term. The model includes year and region-specific time trends which should account for the strength of the anti-vaccination movement at regional level.

The coefficient of interest in this model is *β*, which represents the percentage change in MMR coverage associated with a percentage point change in the real public health expenditure. To test whether our results were driven by potential unobserved heterogeneity we used a within-group estimation. We clustered the standard error by region to reflect non-independent sampling. All models were estimated using STATA version 15.

## Results

**Figure 1:**
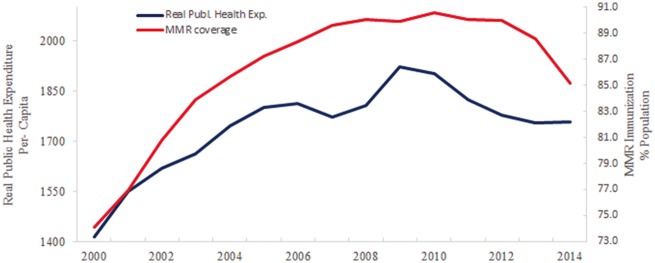
Trends in real public health expenditure per-capita and MMR coverage in Italy, 2000–14 Source: Real public health expenditure per-capita: authors’ elaboration on data from the WHO, Health for All. MMR coverage: Author’s elaboration on data from Superior Health Institute (ISS)

Figure 1 shows that the rising trend in real public health expenditure per-capita reversed, starting from 2010. It should be noted that, until then, it was growing rapidly, at a yearly average of 3.51% from 2000 to 2009 but then it reversed its trend and dropped by about 2% between 2010 and 2014.

These aggregate figures do, however, mask regional variations. In nine regions, real public health spending rose in some years (Liguria, Lombardia, Emilia Romagna, Lazio, Puglia, Calabria, Sardinia, Tuscany). In contrast, the greatest reductions were observed in the Southern, more deprived, regions such as Basilicata and Molise, which experienced declines of over 10%.

**Figure 2:**
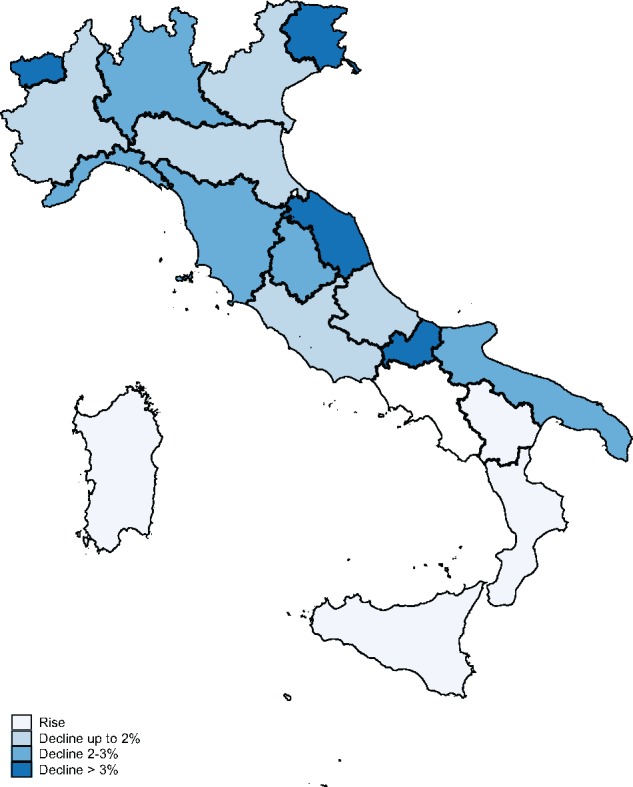
Variation in MMR coverage across regions in Italy, comparison 2010–13. Notes: data for Trentino Alto Adige available at province level only. Source: authors’ elaboration on data from Italian Healthcare Institute

Prior to these reductions, MMR coverage was slowly rising, from 74.1% in 2000 to 90.6% in 2012 (90%) (figure 1). After this period, and coinciding with the introduction of austerity measures, coverage levels fell to 85.1% in 2014. Between 2010 and 2013, in particular, those three regions experiencing the largest drop in MMR coverage (>3%) within the study period were also the ones experiencing the largest financial cuts (Friuli Venezia Giulia—3.39%, Marche—3.59% and Valle d’Aosta—6.89%) (figure 2).

**Figure 3:**
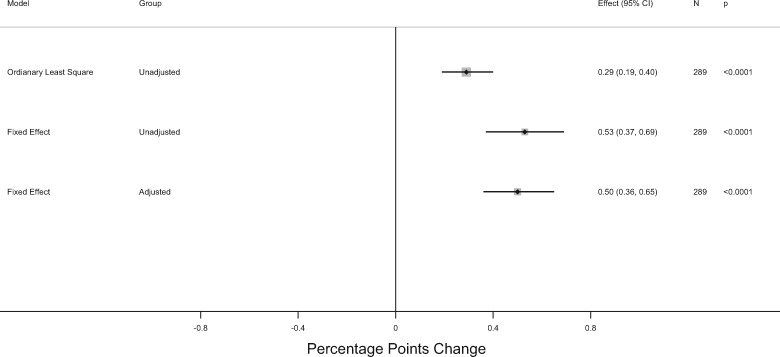
Adjusted association of MMR coverage and real public health expenditure per capita across 20 Italian regions, 2000–14. Confidence intervals are based on robust standard errors, clustered at regional level. Adjusted model control for regional and region-specific time trends. Models are in logs and estimated coefficients represent elasticity

Figure 3 depicts the estimates from a series of statistical models (full results are reported in Supplementary Web Appendix table A2). Our unadjusted model estimates that each 1% increase in real public health expenditure was associated with a significant increase in MMR coverage of 0.29 percentage points (95% CI: 0.19–0.40 percentage points). After controlling for regional fixed effects, to address time-consistent variations in surveillance and health infrastructure, the corresponding figure increased to 0.53 percentage points (95% CI: 0.37–0.69 percentage points). Similarly, when correcting for regional time trends, every 1% increase in real public health expenditure per-capita (corresponding to an average of €17 per-capita) was associated with an increase of 0.50 percentage points in MMR coverage (95% CI: 0.36–0.65 percentage points).

These associations are consistent with the observed regional pattern of measles resurgence. The largest outbreak in Italy was in Lazio, which includes Rome. There, a drop of about 5% in real public health expenditure spending per-capita was associated with a drop of about 2.5% points in MMR coverage. The second largest outbreak was in Piemonte, in the north, also characterized by a drop of over 5% in real public health expenditure per-capita, which experienced a drop of 3% points in the MMR coverage. Interestingly, even in the richer northern regions, the largest declines in MMR coverage appear to coincide with the largest falls in per-capita health expenditure. Examples include Valle d’Aosta, where a fall of more than 6% in spending was associated with a reduction in MMR coverage of more than 11 percentage points and Friuli Venezia Giulia, where per-capita health expenditure fell by over 3%, with a reduction in MMR coverage of about 6.65 percentage points.

Sicily and Sardinia provide a marked contrast to this overall picture. Our data show that Sardinia increased immunization coverage by about 3.8 percentage points and Sicily by about 1.40 percentage points over the period 2010–13. For historical reasons, these two regions, together with Trentino Alto Adige (i.e. Trento and Bolzano), Valle d’Aosta, and Friuli Venezia Giulia, enjoy greater autonomy under the Italian constitution. Sicily and Sardinia are also considered among the most proactive in Italy in terms of immunization policies. The former was the first to introduce varicella immunization in 2003 and the latter was one of the first regions to comply with the 2012–14 National Immunization Plan.^27–29^ During the period covered by the present analyses, Sardinia managed to increase its budget by a yearly average of 3%, and after 2008 by about 2%, the largest rise among all the regions since the beginning of the Great Recession.

### Robustness checks

We conducted a series of robustness checks to the model specification. First, as the outbreaks can be non-linear, we replicated our results using log–log functions, finding consistent patterns (see Supplementary Web Appendix tables A2 and A3). Second, we added the German-speaking province of Bolzano However, none of the main results was qualitatively changed, although the coefficient estimates were slightly attenuated.

## Discussion

Our analysis suggests that austerity measures adopted in Italy contributed significantly to the resurgence of measles. We estimated that each 1% reduction in real public health expenditure per-capita corresponded to a 0.5 percentage points reduction in MMR coverage. In the worst affected regions, which faced up to 5% cuts, this would equate to a 2.5 percentage point fall in coverage. This result is in line with previous studies relating declining pattern in vaccination coverage with economic factors such as poverty,^30^ perceived distance to clinics,^31^ low family income^32–34^ and lack of health insurance.^35–37^

Before analyzing potential implications for policy and research, we must note several limitations of our analysis. First, our study did not evaluate infections, but vaccination rates. There is little doubt that the primary risk factor for measles outbreaks is declining vaccination coverage levels and the population inability to achieve herd immunity rates of 95%. Ideally, we would have liked to further evaluate the impact of reductions in public health expenditures on measles hospitalization and incidence rates. Unfortunately, this would have required a surveillance system with linked data, which are not currently available. Second, our data do not allow us to control for all potential relevant variables and we acknowledge this limitation to our analysis. Third, although fine-grained data on budgets for infectious diseases are available for some regions and years, these are neither routinely recorded nor comparable over time and regions and, thus, have not been used in the current analysis. Moreover, given that even aggregate figures are available only up to 2014, we have not been able to examine the very recent measles outbreak that occurred in Italy in 2017 and produced over 5000 cases among, mostly, unvaccinated individuals. Fourth, the Italian healthcare system is regionally devolved, so that, even though it is a National Health Service, the regions are responsible for financing, planning and implementing healthcare services. This creates diversity across the regions, especially among those five regions that receive greater funding because, historically, were more disadvantaged (specifically, Friuli Venezia Giulia, Trentino Alto Adige, Sicily, Sardinia and Valle d’Aosta). Fifth, although we excluded the province of Bolzano *a priori* because of its specific characteristics, including its extremely low MMR coverage rate, we concede that its data are inconsistent with our finding that public health expenditure is positively correlated with MMR coverage. However, when we run robustness checks to test whether its inclusion affects our overall findings, we find that it does not.

Notwithstanding these limitations, our observations can contribute to understanding the regional patterning of declining measles vaccination coverage in Italy. Importantly, the declines were concentrated neither in the most deprived nor most affluent regions, but in those which experienced the greatest reductions in the real public health expenditure. Thus, even though Sardinia is one of the poorest regions, it increased in some years real public health expenditure per-capita while Italy as a whole was implementing austerity and it did not experience significant measles outbreaks.

In March 2017, WHO expressed concern about the measles outbreaks affecting Europe, despite the availability of a safe, effective vaccine.^38^ In this context, a new law adopted in Italy jn July of that year offers lessons for elsewhere.^39^ This law made vaccination mandatory for children entering public primary schools. However, this has been highly controversial and those opposing vaccination in the Province of Bolzano seek to avoid it through private schooling or crossing borders to Austria and other neighbouring countries. It is still too early to know whether the new immunization plan will be effective. However, early signs are promising, with coverage data presented at the end of December 2017 estimating a national increase from 87.9 to 92.2% at 36 months of age.

The Italian changes have echoes in other European countries. In Germany, while parents have had to show proof that they have received counselling on vaccination before their children can enter kindergarten for the past three years, a recently proposed law would make it mandatory for all kindergartens to notify the health authorities if parents have not submitted such proof. After the introduction of mandatory vaccination in Italy, the French Prime, Minister, Édouard Philippe, stated that ‘it was unacceptable that children are still dying of measles in the country where some of the earliest vaccines were pioneered’.^40^ Currently, diphtheria, tetanus and polio vaccines are mandatory in France. However, the government made eight additional vaccines mandatory from 1 January 2018: whooping cough, measles, mumps, rubella, hepatitis B, influenza, pneumonia and meningitis C.

The most recent Italian budget will increase health expenditure by 1.3% per year in the years 2018–20, which will create fiscal space for investing in infectious disease prevention. However, whether this will be enough to reverse the decline in vaccination coverage remains to be seen, as it remains less than the predicted increase in GDP.

In conclusion, these findings highlight the risks of disinvestment in public health services. Italy is now addressing its low vaccination rate, by a combination of legislation and budgetary increases. It will be important to monitor these developments, not only to inform policy in Italy but across Europe where many countries now face similar problems.

## Supplementary Material

Supplementary Appendix
