# Supplementary material for: Austerity, measles and mandatory vaccination: cross-regional analysis of vaccination in Italy 2000–14
Source: Eur J Public Health. 2018 Sep 11;29(1):123–7. doi: 10.1093/eurpub/cky178 (PMC6345202; doi:10.1093/eurpub/cky178)
Supplement: Supplementary Appendix [file cky178_supplementary_appendix.pdf]

Austerity, measles and mandatory vaccination: cross-regional analysis of vaccination in Italy 2000-2014 - Web Appendix 1

Figure A1: Trends in public healthcare expenditure in preventive care per capita in Italy, 2006-2012.

Figure A2: Estimated association of MMR coverage and real public health expenditure per capita across 21 Italian regions, 2000-2014.

Figure A3 Estimated association of MMR coverage and real public health expenditure per capita across 20 Italian regions, 2000-2014.

Table A1: Descriptive Statistics.

Table A2: Estimated association of MMR coverage and real public health expenditure per capita across 20 Italian regions, 2000-2014

Table A3: Robustness test. Estimated association of MMR coverage and real public health expenditure per capita across 20/21 Italian regions, 2000-2014

Figure A1: Trends in real public healthcare expenditure in preventive care per capita in Italy, 2006-2012.

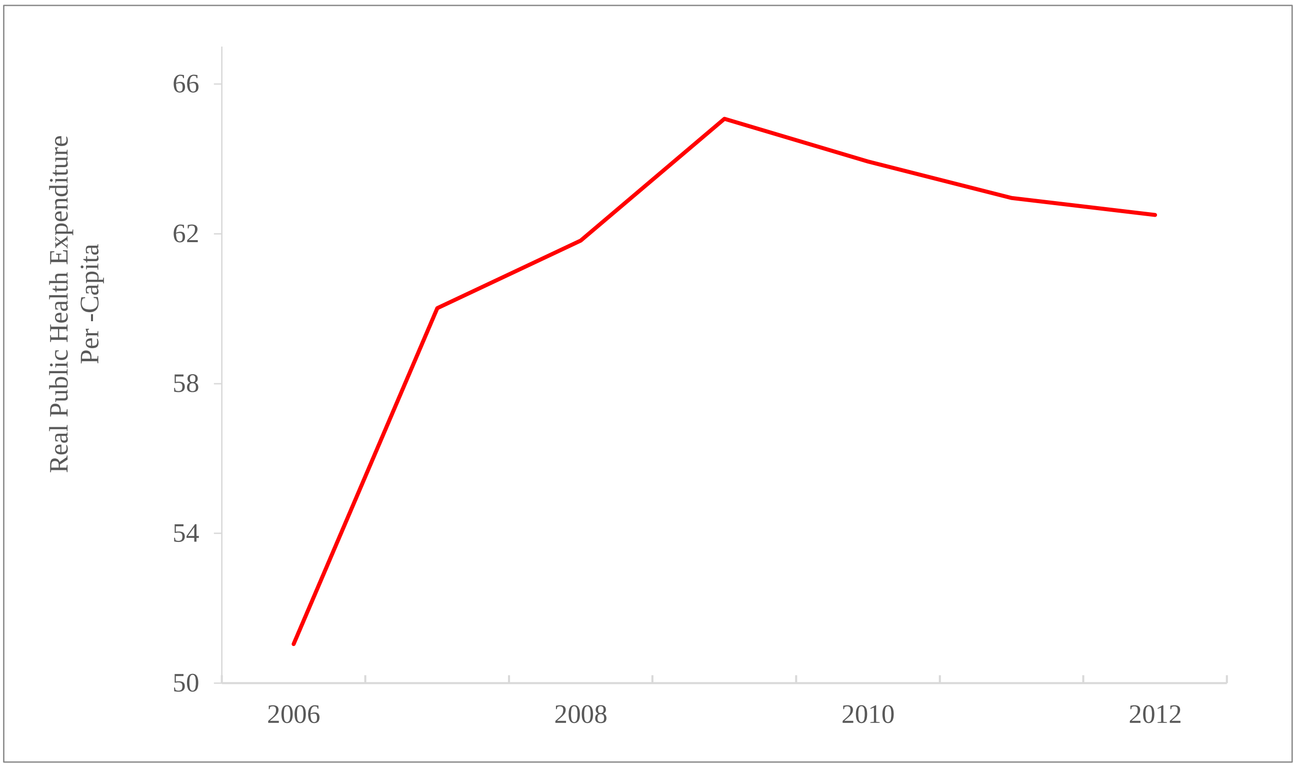

Note: Source: Italian Healthcare Institute[1]

Figure A2: Estimated association of MMR coverage and real public health expenditure per capita across 21 Italian regions, 2000-2014. Confidence intervals are based on robust standard errors, clustered at regional level. Models include the province of Bolzano. Adjusted model control for regional and region-specific time trends.

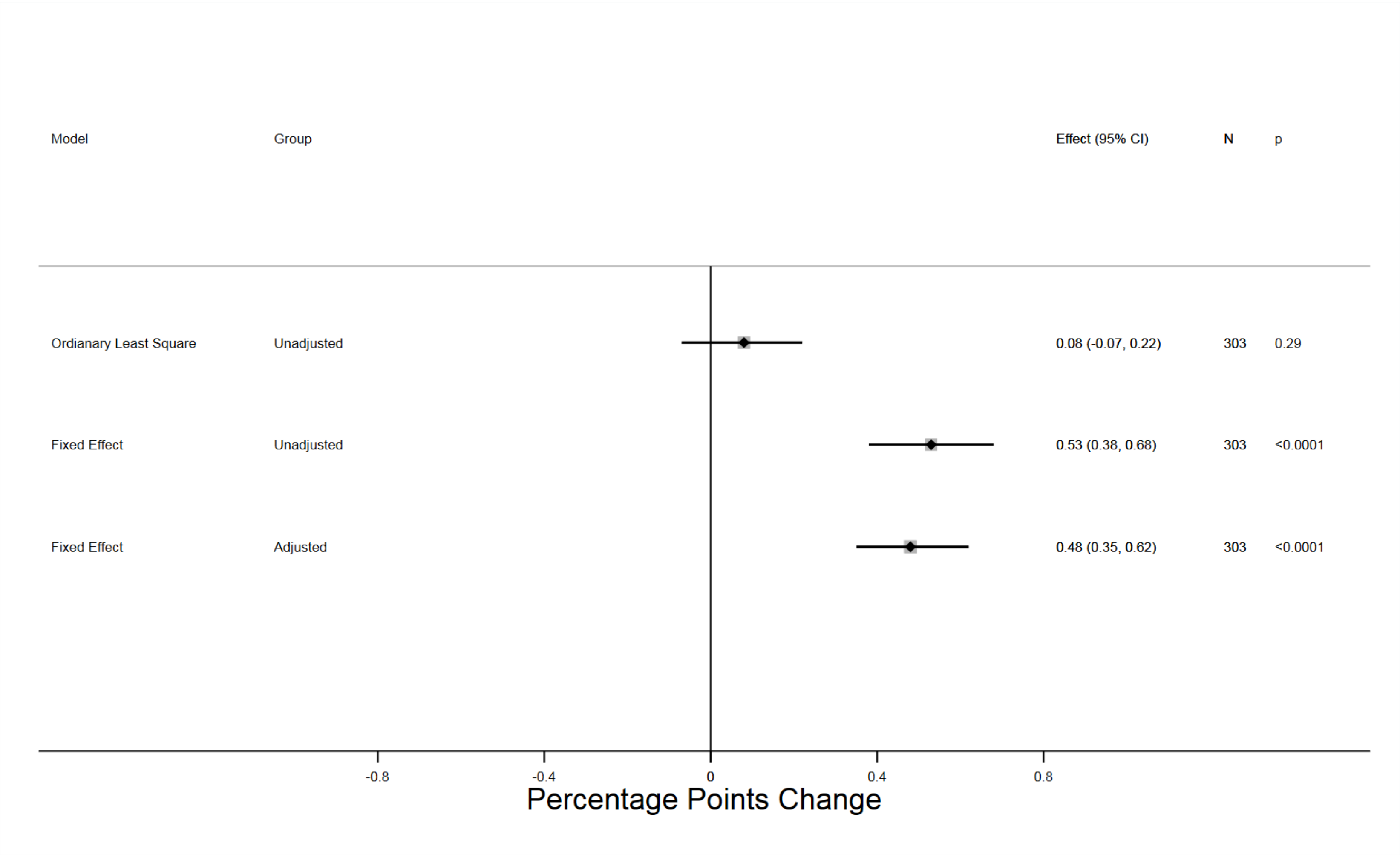

Figure A3: Adjusted association of MMR coverage and real public health expenditure per capita across 20 Italian regions, 2000-2014. Confidence intervals are based on robust standard errors, clustered at regional level. Adjusted model control for regional and region-specific time trends. Models are in logs and estimated coefficients represent elasticity.

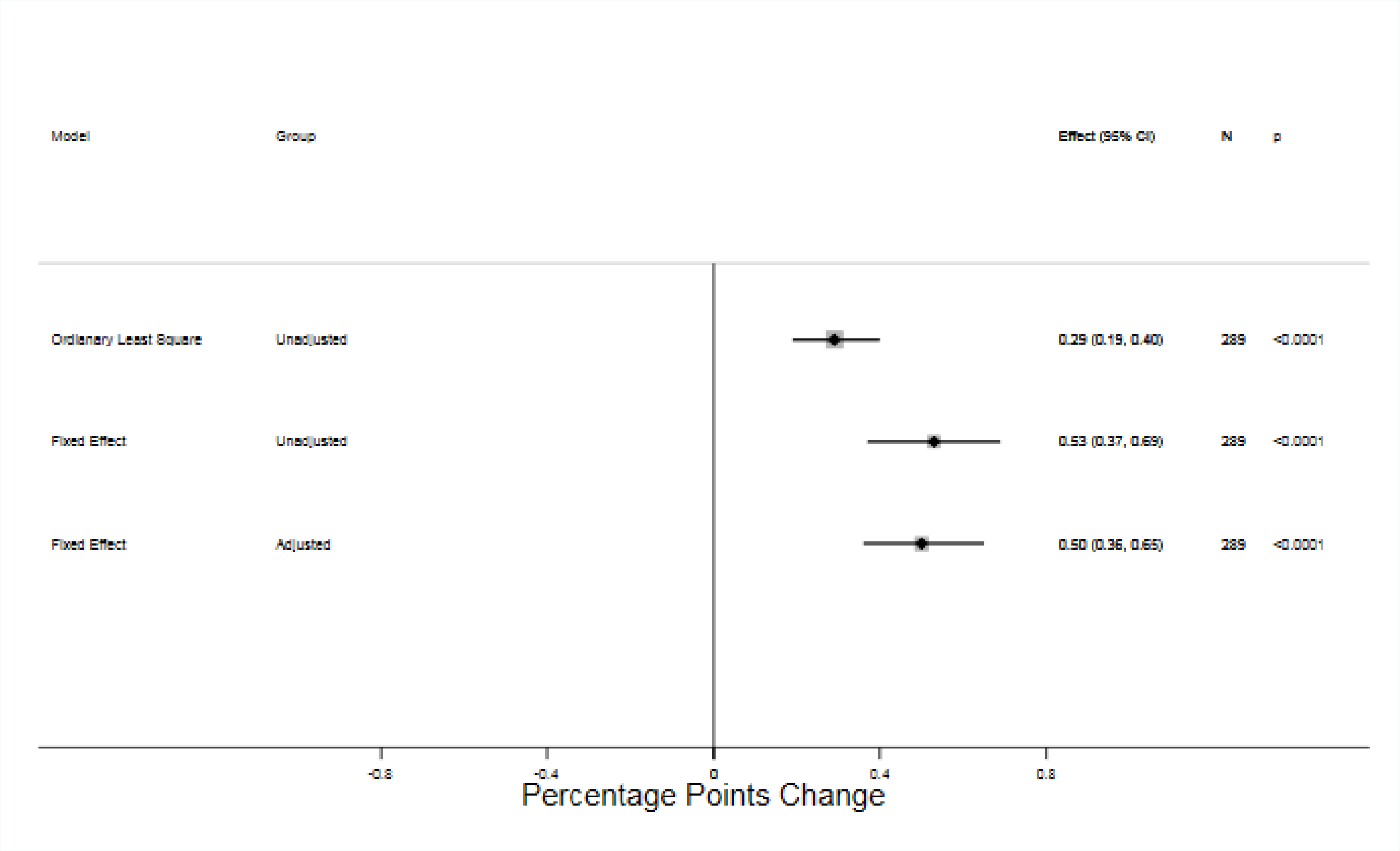

Table A1: Descriptive Statistics

Variable	Number of Region Years	Mean (S.D.)	Min.	Max.	Source
Per-capita Real Health Expenditure Per Year in Euros	300	1741 (178)	1198	2194	HFA
% of Population covered by MMR jab	289	87.5 (7.28)	53	97.3	ISS

Notes: Merged data at Regional level from Health for All (WHO)[1] and Superior Health Institute (ISS)[1] covering the period 2000-2014

Table A2: Estimated association of MMR coverage and real public health expenditure per capita across 20 Italian regions, 2000-2014.

	Percentage Points Change in MMR coverage	Percentage Points Change in MMR coverage	Percentage Points Change in MMR coverage
	OLS	F.E.	F.E.
Per 1 % increase in Per-capita healthcare expenditure	0.29***<br>[0.19, 0.40]	0.53***<br>[0.37, 0.69]	0.50***<br>[0.36, 0.65]
Time-trend	N	N	Y
Regional-year Trends	N	N	Y
Number of Observations	289	289	289

Notes: Source: Data from ISS and Health for All (2000-2014). The dependent represents the yearly change in MMR coverage at Regional and the main explanatory variable represents the yearly change real per-capita healthcare expenditure. Robust standard errors clustered at regional level and for repeated observations.

* $p < 0.05$ ** $p < 0.01$ *** $p < 0.001$

Table A3: Robustness test, Estimated association of MMR coverage and real public health expenditure per capita across 20/21 Italian regions, 2000-2014.

	Percentage Points Change Including Bolzano MMR coverage	Percentage Points Change Including Bolzano MMR coverage	Percentage Points Change Including Bolzano MMR coverage	% Change Model in Logs MMR coverage	% Change Model in Logs MMR coverage	% Change Model in Logs MMR coverage
	OLS	F.E.	F.E.	OLS	F.E.	F.E.
Per 1% increase in Per-capita	0.08*	0.53***	0.48***	0.37***	0.68***	0.64***
healthcare expenditure	[-0.07, 0.22]	[0.38, 0.68]	[0.35, 0.62]	[0.23, 0.52]	[0.47, 0.89]	[0.45, 0.84]
Time-trend	N	N	Y	N	N	Y
Regional-year Trends	N	N	Y	N	N	Y
Number of Observations	303	303	303	289	289	289

Notes: Source: Data from ISS and Health for All (2000-2014). The dependent represents the yearly change in MMR coverage at Regional and the main explanatory variable represents the yearly change real pro-capita healthcare expenditure. Robust standard errors clustered at regional level and for repeated observations.

* $p < 0.05$ ** $p < 0.01$ *** $p < 0.001$

References

1. (ISS) IHI. Coperture Vaccinali MPR 2000-2016, 2017.
